# Soil nutrient amendment increases the potential for inter-kingdom resource competition among foliar endophytes

**DOI:** 10.1093/ismeco/ycae130

**Published:** 2024-10-25

**Authors:** Zoe A Hansen, Michael R Fulcher, Nicholas Wornson, Seth A Spawn-Lee, Mitch Johnson, Zewei Song, Matthew Michalska-Smith, Georgiana May, Eric W Seabloom, Elizabeth T Borer, Linda L Kinkel

**Affiliations:** Department of Plant Pathology, University of Minnesota, Saint Paul, MN 55108, United States; United States Department of Agriculture, Agricultural Research Service, Foreign Disease-Weed Science Research Unit, Frederick, MD 21702, United States; Department of Plant Pathology, University of Minnesota, Saint Paul, MN 55108, United States; Department of Integrative Biology, University of Wisconsin-Madison, Madison, WI 53706, United States; Department of Horticulture, University of Minnesota, Saint Paul, MN 55108, United States; Department of Plant Pathology, University of Minnesota, Saint Paul, MN 55108, United States; Department of Plant Pathology, University of Minnesota, Saint Paul, MN 55108, United States; Department of Ecology, Evolution and Behavior, University of Minnesota, Saint Paul, MN 55108, United States; Department of Ecology, Evolution and Behavior, University of Minnesota, Saint Paul, MN 55108, United States; Department of Ecology, Evolution and Behavior, University of Minnesota, Saint Paul, MN 55108, United States; Department of Ecology, Evolution and Behavior, University of Minnesota, Saint Paul, MN 55108, United States; Department of Plant Pathology, University of Minnesota, Saint Paul, MN 55108, United States

**Keywords:** foliar endophytes, carbon use, nutrient amendment, *Andropogon gerardii*, competitive interactions

## Abstract

Foliar endophytes play crucial roles in large-scale ecosystem functions such as plant productivity, decomposition, and nutrient cycling. While the possible effects of environmental nutrient supply on the growth and carbon use of endophytic microbes have critical implications for these processes, these impacts are not fully understood. Here, we examined the effects of long-term elevated nitrogen, phosphorus, potassium, and micronutrient (NPKμ) supply on culturable bacterial and fungal foliar endophytes inhabiting the prairie grass *Andropogon gerardii*. We hypothesized that elevated soil nutrients alter the taxonomic composition and carbon use phenotypes of foliar endophytes and significantly shift the potential for resource competition among microbes within leaves. We observed changes in taxonomic composition and carbon use patterns of fungal, but not bacterial, endophytes of *A. gerardii* growing in NPKμ-amended versus ambient conditions. Fungal endophytes from NPKμ-amended plants had distinct carbon use profiles and demonstrated greater specialization across carbon sources compared to control plots. Resource niche overlap between bacterial and fungal endophytes also increased with plot nutrient supply, suggesting enhanced potential for inter-kingdom competition. Collectively, this work suggests that soil nutrient enrichment alters how fungal endophyte communities exist in the foliar environment, with potentially significant implications for broad-scale ecosystem function.

## Introduction

Endophytic microbes inhabit the internal tissues of terrestrial plants and are important contributors to global ecosystem processes including nutrient cycling and decomposition [1–4]. The diversity, composition, and resource use characteristics of endophytic microbes can determine their functional impact on larger-scale processes including plant growth and productivity, stress tolerance, and disease resistance [5–9]. Environmental nutrient availability appears to influence below-ground endophyte community diversity and phylogenetic composition [10–12]. However, this effect is unclear for endophytic phyllosphere communities, as elevated nutrient supply did not alter the global network structure among these microbes [13]. In addition, recent evidence suggests that resource competition may not significantly impact phyllosphere bacteria under lab conditions [14], but the role of this competition among fungi and bacteria co-inhabiting the leaf environment is unknown. Therefore, the impact of soil nutrient supply on resource competition among phyllosphere endophytes under field conditions remains a knowledge gap that is particularly important, given ongoing human impacts to global elemental cycles [15].

Soil nutrient supply is often significantly elevated by anthropogenic activities including inorganic fertilizer use and fossil fuel combustion [16–18], which is associated with increased plant productivity [19] but reductions in plant species diversity and temporal stability of community composition and biomass [20, 21] with significant consequences to ecosystem function [22]. More specifically, elevated nutrients can negatively impact soil bacterial and fungal diversity [23, 24], nutrient cycling [25–27], and the complexity of microbial networks [28]. And while elevated soil nutrient supply has been shown to both increase and decrease foliar endophyte diversity [29, 30], the direct and indirect effects of nutrient addition on the functions and interactions of foliar endophytes are not well understood.

Elucidating the consequences of anthropogenic NPK addition on functional relationships between endophytic microbes may improve our understanding of the factors determining foliar community assembly. Plant host phylogeny [31] and environmental variables such as temperature and rainfall [32–34] can alter endophyte community assembly, but the major roles of within-plant competitive species interactions in endophyte community assembly have been poorly described. Soil microbial communities subjected to long-term nutrient deposition demonstrate altered assembly patterns and develop greater functional homogeneity compared to ambient conditions, which occurs at the apparent cost of microbial diversity [35]. In foliar endophytic communities, elevated nutrients have been associated with shifts in the relative abundances of distinct phylogenetic assemblages of slow- and fast-growing fungal endophytes of *A. gerardii* [36] and phylogenetic overdispersion [37], a pattern associated with competitive exclusion among closely related taxa. Indeed, competitive interactions between endophytes within leaf resource niches may strongly affect endophyte assembly [38] through several mechanisms including competitive release [39] and modulation of plant chemistry [40–42]. Resource competition is expected to play a similarly important role in endophyte community assembly as it does in shaping plant-associated soil microbial communities [43] and may also be mediated by exchange of secondary metabolites [44]. Such inhibitory interactions can occur across kingdoms and can be related to the degree of niche overlap [45] as well as plant host species and community richness [46, 47]. Clarifying the effects of nutrient enrichment on the potential for competitive interactions among endophytes is pertinent to understanding foliar community assembly and function.

We examined the effects of long-term NPK and micronutrient (NPKμ) addition on community composition and carbon use phenotypes of bacterial and fungal endophytes inhabiting a ubiquitous native prairie grass, *A. gerardii*. We hypothesized that (i) NPKμ amendment will shift endophytes’ resource use niches and overall growth patterns resulting in altered niche overlap and apparent resource competition among bacteria and fungi and (ii) correspond with compositional and phylogenetic shifts among foliar endophytes. This work provides critical insight into the pervasive, non-target impacts of anthropogenic nutrient deposition on microbial communities, including changes in microbial species interactions and community assembly processes among foliar endophytes.

## Materials and methods

### Isolate collection

Isolates were collected from field experiment plots at Cedar Creek Ecosystem Science Reserve (CCESR), a U.S. National Science Foundation Long Term Ecological Research (LTER) Site in East Bethel, MN. Rainfall at the site averages 750 mm yr^−1^ and the mean annual temperature is 6.3°C. This research was conducted within the Nutrient Network (https://nutnet.org/), a coordinated effort to research the ecological impacts of nutrient deposition on temperate and tropical grasslands [48]. Experimental plots were 5 × 5 m in size and separated by 1 m walkways. Since 2008, these plots have either received annual addition of several soil nutrients or have been maintained as non-treated control plots in a randomized complete block design with three replicate blocks. The soil nutrient treatment (NPKμ) included application of 10 g N m^−2^ yr^−1^ as time-release urea, 10 g P m^−2^ yr^−1^ as triple super phosphate, and 10 g K m^−2^ yr^−1^ as potassium sulfate with an additional initial amendment of micronutrients (100 g m^−2^ of Ca 6%, Mg 3%, S 12%, B 0.1%, Cu 1%, Fe 17%, Mn 2.5%, Mo 0.05%, Zn 1%) in 2008. Two randomly-chosen control plots and two randomly-chosen NPKμ-amended plots were included as representative plots in this study. *A. gerardii,* a native perennial C4 prairie grass, served as a focal plant species for this study due to its predominance in prairie ecosystems. In September 2015, a single mature, non-senescent leaf was collected from six plants within each soil nutrient supply condition, resulting in twelve total leaves (1 leaf × 3 plants × 2 plots/treatment × 2 treatments). Leaves were stored on ice during transport and were subsequently surface-sterilized by submerging for 60 s each in sterile deionized water, 75% ethanol, 0.41% sodium hypochlorite, 75% ethanol, and sterile deionized water. Sterilized leaves were cut into three equally sized sections and a single random section was used for microbial isolation.

Individual leaf sections were macerated for 3 min in 10 ml PBS buffer using an FSH 125 hand-held homogenizer (Fisher Scientific™). Macerate and dilutions [10^−2^, 10^−4^, and 10^−6^] were spread over Petri dishes containing artificial growth media including malt extract agar, water agar, starch-casein agar, and pentachloronitrobenzene peptone agar. Microbes were sub-cultured as they appeared during incubation at room temperature or 28°C. Fungi and bacteria were selected at random from each media, sub-cultured repeatedly until pure strains were obtained, and stored at −80°C in 20% glycerol stock suspensions (bacteria) or on agar slants at 4°C (fungi). In total, 800+ isolates were collected from the consortium of twelve leaves (*n* = 6 control, *n* = 6 NPKμ-amended). From this collection, 10 bacteria and 10 fungi were randomly selected from each leaf for use in this study using a random number table (*n* = 60 bacterial and 60 fungal isolates from control plots and *n* = 60 bacterial and 60 fungal isolates from NPKμ-amended plots). One leaf yielded only five culturable bacterial isolates, resulting in a final total of 115 bacterial and 120 fungal isolates. 16S (bacteria) and ITS (fungi) regions were amplified with PCR and sequenced via capillary array-based Sanger Sequencing. Taxonomic assignments for all isolates were determined from partial gene sequence homology at the 16S (bacteria) [49] and ITS (fungi) [50] loci determined by pairwise alignment to known reference sequences deposited in NCBI GenBank using the Blastn algorithm [51].

### Measuring carbon substrate utilization

For every isolate included in this study (*N* = 235), carbon use was determined using Biolog SF-P2 plates (Biolog, Hayward CA) as described previously [46]. Briefly, each isolate was inoculated into a 96-well microplate containing 95 single-carbon sources and one water control. Plates were incubated for 72 h at 28°C, followed by measurement of well absorbance at 590 nm. Ability to use a carbon source was defined as an optical density (OD) ≥ 0.005 following subtraction of the absorbance value for the water control from the observed OD for a specific substrate [46, 52, 53]. Carbon use profiles for each isolate were described using three metrics: niche width, total growth, and growth efficiency (Equations 1–3):


(1) \begin{equation*} \mathrm{Niche}\ \mathrm{Width}\ \left(\mathrm{NW}\right)={N}_{\mathrm{substrates}\ \mathrm{consumed}} \end{equation*}

(2) \begin{equation*} \mathrm{Total}\ \mathrm{Growth}\ \left(\mathrm{TG}\right)=\sum{\mathrm{OD}}_{\mathrm{substrates}\ \mathrm{consumed}} \end{equation*}

(3) \begin{equation*} \mathrm{Growth}\ \mathrm{Efficiency}\ \left(\mathrm{GE}\right)={\overline{\mathrm{OD}}}_{\mathrm{substrates}\ \mathrm{consumed}}=\frac{\mathrm{Total}\ \mathrm{Growth}}{\mathrm{Niche}\ \mathrm{Width}} \end{equation*}

The growth efficiency metric was used to formulate a resource niche for each isolate. Relative niche overlap (NO) was calculated for each pairwise combination of isolates (Equation 4):


(4) \begin{equation*} {\omega}_{i\to j,n}=\min \left(\frac{{\mathrm{OD}}_{i,n}}{{\mathrm{OD}}_{j,n}}\right) \end{equation*}

NO was calculated for each of the *m*(*m −* 1) ordered pairs of isolates *i* and *j* (where *m* is the number of unique isolates) and for each substrate *n* on which both isolates demonstrate non-zero growth. The term OD*_i,n_* indicates the optical density of isolate *i* after 72 h of growth on carbon source *n* and refers to the fraction of isolate *j*’s growth on substrate *n* that is matched by isolate *i*. These values were averaged across substrates to obtain a single value for each pair of isolates (Equation 5):


(5) \begin{equation*} {\overline{\omega}}_{i\to j}=\frac{1}{95}\sum_{n=1}^{95}{\omega}_{i\to j,n} \end{equation*}

Pairwise NO values were considered a proxy for competitive interactions among isolates, with greater NO values suggesting stronger competition. This metric is directional and therefore two values are represented within a single pair of isolates (Supplementary Fig. S1). These values are reported from a particular isolate’s (*i* or *j*) “perspective,” hence the directional nature of the metric. “Outgoing” niche overlap for an isolate, *i,* represents the proportion of another isolate’s, *j*, resource niche that its own resource niche overlaps (*i → j)*. Reciprocally, outgoing niche overlap for isolate *j* is the proportion of isolate *i*’s resource niche that *j’s* resource niche overlaps (*j → i)*. These values are not always symmetrical, as the resource niches for isolate *i* and isolate *j* may differ in the number of substrates consumed (niche width) as well as the degree to which these substrates are consumed (total growth) which fundamentally alters their resource niche. Asymmetry in niche overlap values between isolate *i* and isolate *j* suggests that two isolates are impacted differently by a single competitive interaction event, with one isolate potentially experiencing greater resource costs of competition than the other.

### Statistical analysis

Statistical analyses were conducted in RStudio version 1.1.463 (RStudio Development Team 2016) using R version 4.04 and the vegan package (version 2.5-7) [54] unless otherwise noted. The 2-Sample Test of Equal Proportions was used to compare taxonomic relative abundance between control and NPKμ-amended plots, with a significance cutoff of α = 0.05. A Welch’s *t*-test (for normally distributed data) or Wilcoxon rank-sum tests (for non-normally distributed data) were used to assess carbon use evenness and resource niche overlap between control and NPKμ-amended plots, and between Fungi and Bacteria within a particular treatment. Normality of data was determined using a Shapiro–Wilk normality test (“shapiro_test” from the rstatix package (version 0.7.0)). Multiple hypothesis correction was performed using the Benjamini–Hochberg method with a false discovery rate (FDR) of 0.05.

Niche width, total growth, and growth efficiency were analyzed using generalized linear models that included a fixed effect interaction between kingdom and nutrient treatment (control vs. NPKμ-amended) and a random effect for leaf ID. Analysis of variance and *R*^2^ values were used to assess predictor significance and model fit. Least-squares means of kingdom and nutrient treatment were contrasted at α = 0.05. Niche width was modelled assuming a Poisson distribution while growth efficiency and total growth were log-transformed to meet assumptions of normality.

Carbon use dissimilarity matrices were constructed using the Bray–Curtis dissimilarity of carbon use profiles for all isolates (absolute absorbance values across all 95 single-carbon compounds were considered following subtraction of the water control). Carbon use profiles were contrasted between kingdoms and treatments using non-metric multidimensional scaling (NMDS) analysis and permutational analysis of variance (PERMANOVA) using the adonis2 function from the vegan package in R. This was followed by permutational analysis of multivariate dispersions (PERMDISP) using the betadisper function with 9999 permutations. Pairwise differences between kingdoms and treatments were assessed using Tukey’s honestly significant difference test at α = 0.05.

Phylogenetic distances for all isolates were calculated separately for bacterial and fungal isolates, using 16S sequences and ITS sequences, respectively. Sequences were aligned using MUSCLE [55] and alignments were used to (i) create a maximum likelihood phylogenetic tree of all cultured isolates using RAxML (v8.2.11) with 1000 replicate trees [56] and (ii) generate a matrix of pairwise phylogenetic distances in MEGAX version 11.0.13. Kingdoms were considered separately when generating phylogenies. Correlations between phylogenetic distance and carbon use dissimilarity were examined using a Mantel test from the vegan package with 999 permutations and Pearson correlation of pairwise measures with a cutoff of α = 0.05. Additionally, we performed linear modeling to observe the relationship between carbon use dissimilarity, phylogenetic distance, treatment, and kingdom with a confidence interval of 0.95.

## Results

### NPKμ amendment alters community composition among fungal but not bacterial endophytes

**Figure 1:**
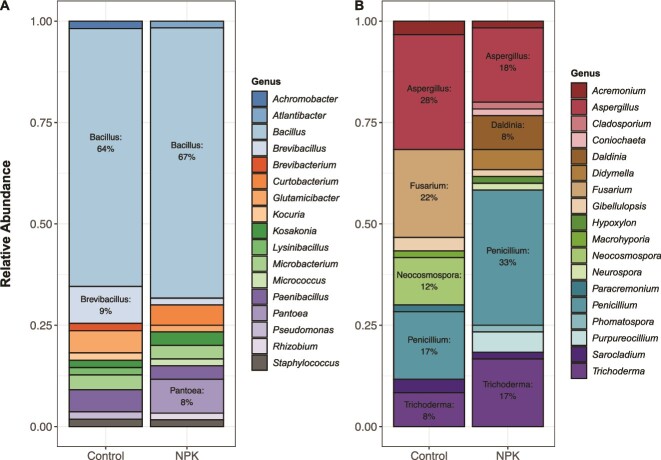
Changes in community composition of foliar endophytes are more apparent in sampled fungal, not bacterial, isolates. Relative abundances of 115 bacterial (A) and 120 fungal (B) foliar endophytes are shown based on 16S (bacteria) and ITS (fungi) sequencing identification, respectively. Taxonomic assignments are shown at the genus level for both the control and NPKμ-amended treatments. Bacterial and fungal genera comprising the highest proportions of relative abundance are noted within the stacked barplots.

Taxonomic composition of culturable fungal endophytes (*n* = 120) appeared to differ between control and NPKμ-amended plots, but bacterial endophytes (*n* = 115) did not demonstrate this difference in community composition between plots. However, variance among bacterial and fungal composition across leaves did not differ between treatments (Supplementary Fig. S2), and there was no statistically significant difference in fungal composition between plots (PERMANOVA; *F* = 1.44, *P* = .254). Seven bacterial genera were observed in both control and NPKμ-amended leaves, while six fungal genera were found across both plot conditions (Supplementary Table S1; Fig. 1). Among bacterial genera, *Bacillus* comprised a majority of isolates across both conditions (64% of isolates in control plots, 67% of isolates from amended plots). Of the fungal genera collected from both plot conditions, *Aspergillus* comprised 28% of isolates obtained from control leaves, while accounting for only 18% of fungal isolates in NPKμ-amended leaves (2-Sample Test of Equal Proportions, χ^2^ = 1.16, *P* = 0.195). In contrast, *Penicillium* increased in relative abundance from 17% to 33% and *Trichoderma* increased from 8% to 17% in control versus NPKμ-amended leaves, respectively (2-Sample Test of Equal Proportions, χ^2^ = 3.60, p_Penicillium_ = 0.058; χ^2^ = 1.22 p_Trichoderma_ = 0.270). Additionally, five bacterial genera and four fungal genera were obtained only from leaves in control plots, while five bacterial genera and eight fungal genera were collected exclusively from NPKμ-amended conditions. These differences in the relative abundances of foliar fungi were most striking between plot conditions. The four fungal genera that were collected exclusively from control plots included *Fusarium* (22% of isolates from control plots) and *Neocosmospora* (12%). The eight genera exclusively found in NPKμ-treated plots included *Daldinia* (8% of isolates from NPKμ-amended plots), *Didymella* (5%), and *Purpureocillium* (5%). The absence of isolates in leaves of an experimental treatment does not confirm their absence *in planta*, however. This work’s focus on culturable microbes complements previous works that investigated composition of the entire leaf community of these *A. gerardii* leaves via amplicon sequencing analysis [29, 37]. These studies demonstrate moderate concordance with our identification of the various bacteria and fungi present in our cultured set of microbes. With regard to fungi, amplicon sequencing analysis showed a high relative abundance of fungi in the class Dothideomycetes. However, many of the fungi cultured in our study are Sordariomycetes or Eurotiomycetes, which were indeed present but in lower relative abundance in these previous amplicon studies. Alternative culturing techniques completed on leaves of *A. gerardii* did identify members of Dothideomycetes, but many of these isolates were slow-growing, while cultured isolates from Sordariomycetes grew notably faster [36]. Thus, our subset of culturable isolates is at least partially representative of the whole leaf environment, with potential bias toward faster-growing fungi. Since all isolates within our subset were cultured under the same conditions when assessing endophytic phenotypes, however, comparisons within our subset are valid. Overall, the observed shifts in culturable fungal, but not bacterial, taxonomic composition shown here suggest that fungal endophyte communities were more responsive to NPKμ amendment than bacterial communities.

### NPKμ amendment alters carbon consumption patterns among foliar endophytes

**Figure 2:**
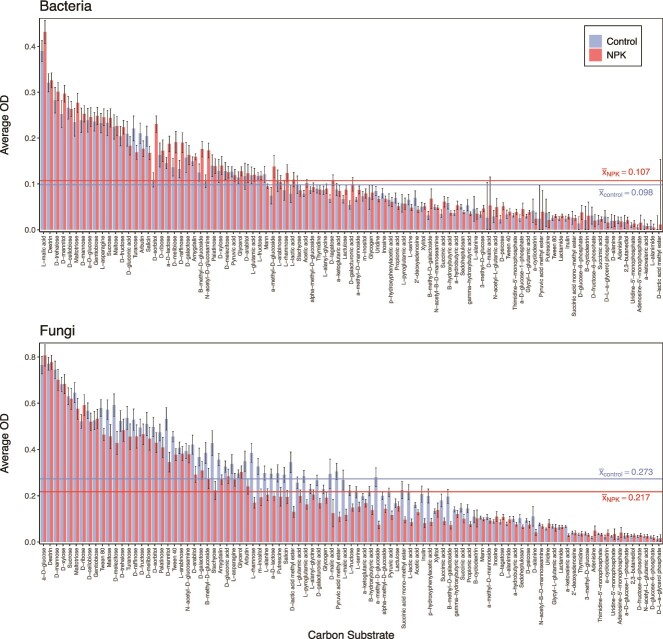
NPKμ amendment augments growth of bacterial, but not fungal, endophytes across 95 single-carbon sources. Average optical density (OD) on each of the 95 single-carbon sources (bars) is shown for bacterial (top) and fungal (bottom) isolates from control and NPKμ-amended conditions. The overall mean OD values across bacteria or fungi within a treatment are displayed as horizontal lines. Carbon sources on the x-axis are plotted in descending order based on average OD; this order differs for bacteria and fungi. Standard error of the mean OD across isolates within a treatment is shown for each nutrient; error bars that extended to a negative value were truncated at y = 0.

Of 95 single-carbon sources, every C substrate was used by at least nine of the 235 microbial endophytic isolates, while none of the substrates were utilized by all bacterial and fungal endophytic isolates. We observed a wide range of growth (OD) across the 95 substrates (OD 0.018–0.553 absorbance at 590 nm) (Fig. 2). Fungal isolates had significantly greater mean OD values across all carbon sources compared to bacteria (x̅_F_ = 0.245 vs. x̅_B_ = 0.103; Wilcoxon rank-sum test: *P* < .0001). While bacterial endophytes from NPKμ-amended leaves had greater growth than those from control leaves, this difference was not significant (x̅_control_ = 0.098, x̅_NPKμ_ = 0.107; *P* = .449). In contrast, fungi from control plots had significantly greater mean growth than those from NPKμ amendment (x̅_control_ = 0.273, x̅_NPKμ_ = 0.217; *P* = .0413).

**Figure 3:**
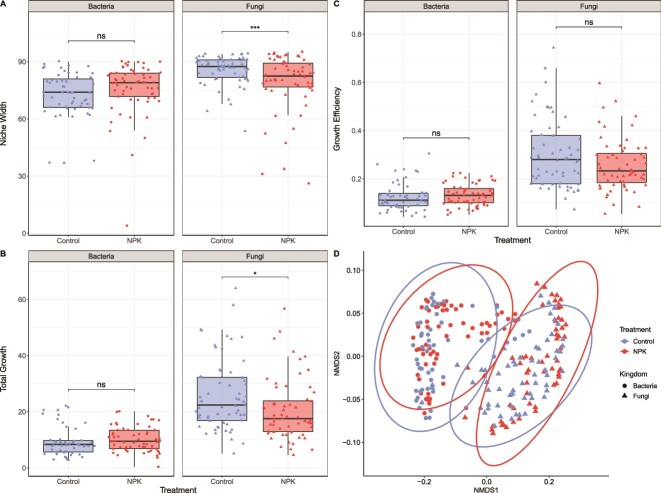
NPKμ amendment is associated with changes in nutrient use among bacterial and fungal endophytes. Faceted boxplots demonstrate shifts in the estimated marginal means of niche width (A), total growth (B), and growth efficiency (C) of bacterial and fungal isolates from control and nutrient-amended plots. Each box displays the median (thick, black line) and upper and lower quartile values shown as the upper and lower limits of the box, respectively. Estimated marginal means were compared and significant differences are shown based on α = 0.05 with significance cutoffs as follows: <.001 “^***^”; <.01 “^**^”; <.05 “^*^”; >.5 “ns”. (D) Nonmetric multidimensional scaling (NMDS) plot demonstrating differences in carbon use profiles among bacterial (circles) and fungal (triangles) endophytes from control (blue) and NPKμ-amended (red) plots. Points are oriented based on the Bray–Curtis dissimilarity of nutrient use across 95 single-carbon sources. Ellipses indicate the 95% confidence interval for the multivariate *t*-distribution of each group.

Overall, carbon use of fungal but not bacterial endophytes differed between microbes from NPKμ-amended and control plots. The ten substrates supporting the greatest mean growth (“preferred substrates”) among all bacteria did not differ between NPKu and non-amended conditions. However, among all fungi, those isolated from control and NPKμ-amended plots shared 7 of 10 preferred substrates (Supplementary Table S2). Reduced niche width, total growth, and growth efficiency were observed among fungi from NPKμ-amended compared to those from control conditions (Fig. 3A–C). Bacteria from NPKμ-amended plots demonstrated only marginal increases across all three metrics and did not differ significantly from bacteria collected from control plots. In contrast, fungal isolates from NPKμ amendment plots had significantly smaller niche widths than control plots (${\overline{\mathrm{NW}}}_{\mathrm{F}-\mathrm{control}}$=85.5 vs. ${\overline{\mathrm{NW}}}_{\mathrm{F}-\mathrm{NPK}\mathrm{\mu}}$=79.2; *P* = .0001). In a similar manner, total growth, which measures the cumulative optical density across all 95 carbon sources, was lower among fungal endophytes from NPKμ-amended leaves versus control leaves (${\overline{\mathrm{TG}}}_{\mathrm{F}-\mathrm{control}}$=23.1 vs. ${\overline{\mathrm{TG}}}_{\mathrm{F}-\mathrm{NPK}\mathrm{\mu}}$=18.1; *P _=_* .0102). Notably, these negative shifts in niche width and total growth demonstrated by fungi were observed among fungal genera present in leaves collected from both control and NPK-amended plots (Supplementary Fig. S3). These findings show that soil NPKμ amendment affects the fungal taxonomic composition of endophyte communities and is associated with shifts in their carbon use phenotypes. Interestingly, growth efficiency, which measures mean growth on all *utilized* substrates, did not differ significantly between treatments for bacterial (${\overline{\mathrm{GE}}}_{\mathrm{B}-\mathrm{control}}$=0.116 vs. ${\overline{\mathrm{GE}}}_{\mathrm{B}-\mathrm{NPK}\mathrm{\mu}}$=0.125, *P* = .389) or fungal endophytes (${\overline{\mathrm{GE}}}_{\mathrm{F}-\mathrm{control}}$=0.271 vs. ${\overline{\mathrm{GE}}}_{\mathrm{F}-\mathrm{NPK}\mathrm{\mu}}$=0.235, *P* = .0535). However, fungi had significantly greater growth efficiency than bacteria regardless of treatment (*P* < .0001).

Additionally, fungal and bacterial endophytes clustered separately based on their carbon use profiles (Fig. 3D) and, notably, these profiles were more dissimilar between kingdoms (e.g. fungi vs. bacteria) than between treatments within a kingdom (e.g. B_control_ vs. B_NPKμ_). Indeed, permutational analysis of variance (PERMANOVA) demonstrated that kingdom explained the greatest proportion of variance among carbon use dissimilarity (*R*^2^ = 0.516; *P* = .001), with the interaction term between kingdom and treatment having a significant but minor effect (*R*^2^ = 0.01; *P* = .015). Among solely bacterial endophytes, we observed small effects with significant differences in carbon use between isolates collected from control and NPKμ-amended plots (PERMANOVA; *R*^2^ = 0.026; *P* = .049), but no difference in variance across these groups (PERMDISP; *P* = .197). There was also a small effect of treatment and a significant difference in the carbon use profiles of endophytic fungi from control versus NPKμ-amended plots (PERMANOVA; *R*^2^ = 0.040; *P* = .018). Fungi from NPKμ-amended plots demonstrated a larger average distance to the median (0.305) than those from control plots (0.266), suggesting greater variance in carbon use among endophytic fungi from elevated soil nutrient conditions (PERMDISP; *P* = .021).

### Growth strategies differ with nutrient supply and affect apparent cross-kingdom competition for C substrates among foliar endophytes

**Figure 4:**
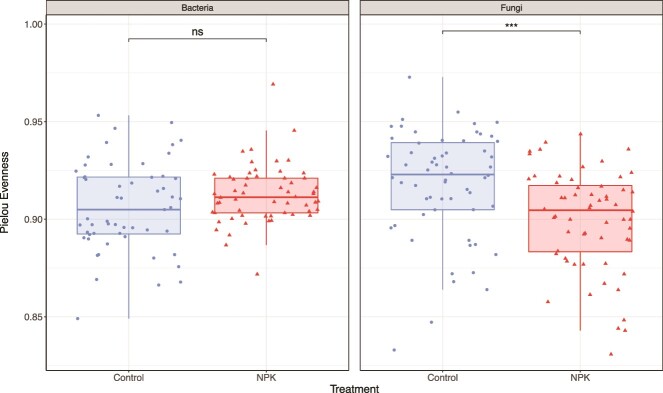
Changes in nutrient use evenness of foliar endophytes are apparent in sampled fungal, but not bacterial, isolates when grown on 95 single-carbon sources. Boxes show the interquartile range of evenness scores (filled region) as well as the median of each group (thick bar in each box). Minima and maxima are indicated by the lower and upper limits of the vertical lines appended to the bottom and top of each box, respectively. Two outliers were removed from the set of bacterial isolates collected from NPKμ-amended plots when performing statistical comparisons and plotting. A Wilcoxon rank-sum test was used to determine significance of differences between treatments within each kingdom: (B_control_ vs. B_NPKμ_, *P* = .073; F_control_ vs. F_NPKμ_, *P* = .000195). Points are jittered to improve visual interpretation. Note that the y-axis scale ranges from approximately 0.80 to 1.0 and does not comprise the full range of Pielou’s evenness index (typically 0.0–1.0).

We measured the evenness of total growth across all 95 C substrates to infer whether isolates displayed generalist or specialist strategies in control and NPKμ-amended conditions. In this context, isolates demonstrating generalist patterns would show greater evenness and homogeneity of carbon use (i.e. similar OD across all C sources consumed) compared to those displaying specialist patterns, which would show more heterogenous carbon use (i.e. greater discrepancy in degree of use for the most- and least-consumed substrates). Bacterial endophytes in NPKμ-amended leaves did not differ in evenness than those in control conditions (x̅_control_ = 0.906, x̅_NPKμ_ = 0.913; Wilcoxon rank-sum test: *P* = .073). Fungi, however, showed significantly lower carbon use evenness in NPKμ-amended leaves compared to control leaves (x̅_control_ = 0.918, x̅_NPKμ_ = 0.900; *P* < .0001) (Fig. 4). Greater evenness in carbon use suggests that fungal endophytes from control conditions employ a more generalist strategy of carbon consumption while fungal isolates originating in NPKμ-amended leaves demonstrate a specialist strategy (Supplementary Fig. S4). These findings suggest that NPKμ amendment may alter growth strategies among endophytic fungi, resulting in greater specialization across carbon sources.

**Figure 5:**
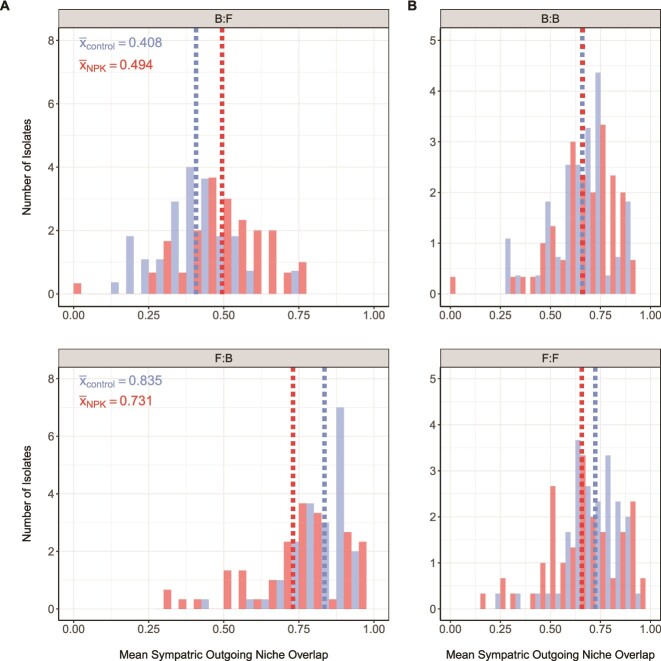
Bacterial competitive capacity against fungi is enhanced in NPKμ conditions. Histograms demonstrate the frequency of niche overlap values for Bacteria:Fungi (B:F) pairings (A-top) and Fungi:Bacteria (F:B) pairings (A-bottom) in control and NPKμ-amended conditions. Niche overlap is a directional measure of the level of resource competition between pairs of isolates based on their nutrient use profiles determined using Biolog SF-P2 plates. The mean outgoing niche overlap values are shown for isolates from NPKμ-amended (red, dotted line) and control (blue, dotted line) plots. These mean values are reported as text within each graph. Notably, B:F overlap is significantly greater (*P* = .000791) and F:B niche overlap is significantly lesser (*P* = .00018) in NPKμ-amended conditions compared to ambient conditions. (B) Intra-kingdom niche overlap values are shown for Bacteria:Bacteria (B:B; top) and Fungi:Fungi (F:F; bottom) pairings from control (blue) and NPKμ-amended (red) conditions. Outgoing niche overlap does not significantly change among B:B (*P* = .821) or F:F (*P* = .071) interactions between control and NPKμ amendment.

Differences in growth across all C substrates for bacterial and fungal endophytes suggest shifts in cross-kingdom resource competition under NPKμ addition. Niche overlap, a directional metric that estimates the intensity of resource competition between co-occurring isolates collected from the same leaf, was used to infer the strength of intra- and inter-kingdom resource competition within leaves. As a reminder, outgoing niche overlap describes one isolate’s (*i*) overlap onto another paired isolate’s (*j*) resource niche, and serves as a proxy for isolate *i’s* competitive ability against *j.* We observed greater outgoing niche overlap among sympatric (within-leaf) Bacteria:Fungi (B:F) interactions (*n*_control_ = 550; *n*_NPKμ_ = 600) in leaves from NPKμ-amended plots than in control plots (x̅_B:F-control_ = 0.408, x̅_B:F-NPKμ_ = 0.494; Welch’s *t*-test: *P* = .000791) (Fig. 5A). At the same time, outgoing niche overlap among Fungi:Bacteria (F:B) pairings decreased in NPKμ plots versus control plots (x̅_F:B-control_ = 0.835, x̅_F:B-NPKμ_ = 0.731; Wilcoxon rank-sum test: *P* = .00018). Collectively, these findings suggest that NPKμ amendment is associated with greater competitive capacity among bacteria against fungi and diminished competitive capacity among fungi against bacteria.

Intra-kingdom pairs were then assessed for shifts in outgoing niche overlap. Intra-kingdom pairs include sympatric bacteria against bacteria (B:B) and fungi against fungi (F:F) within the same leaf. Neither B:B nor F:F pairs demonstrated significant differences in outgoing niche overlap between NPKμ and control plots (x̅_B:B-control_ = 0.660, x̅_B:B-NPKμ_ = 0.660; Wilcoxon rank-sum test: 0.821) or F:F pairs (x̅_F:F-control_ = 0.724, x̅_F:F-NPKμ_ = 0.656; Wilcoxon rank-sum test: 0.071) (Fig. 5B). Together, the results for carbon use evenness and niche overlap suggest that chronically elevated nutrients can increase substrate specialization among endophytic fungi and increase inter-kingdom competition of bacteria against co-occurring fungi.

### Phylogenetic distance is correlated with differences in carbon use among fungi in control but not NPKμ-amended plots

As shown, isolates from NPKμ-amended plots demonstrated differences in resource niche overlap and inferred inter-kingdom competition compared to isolates from control plots. We sought to understand whether these shifts in microbial carbon use phenotype were associated with differences in phylogenetic diversity among endophytes. Firstly, we confirmed that the subset of microbes randomly selected for this study were notably representative of all endophytes collected, cultured, and sequenced from the twelve leaves which were sampled (Supplementary Fig. S5). Importantly, investigating phylogeny in addition to phenotype strengthens our interpretation of how these phenomena may be interacting during endophyte community assembly and how their relationship changes with NPKμ amendment. If phylogenetic diversity is correlated with endophytic communities’ resource use patterns, we may expect to see a strong relationship between phylogenetic distance and carbon use phenotypic distance among isolates regardless of soil nutrient supply. If resource consumption phenotypes are more important during community assembly, however, we may observe little to no relationship between phylogeny and phenotype.

**Figure 6:**
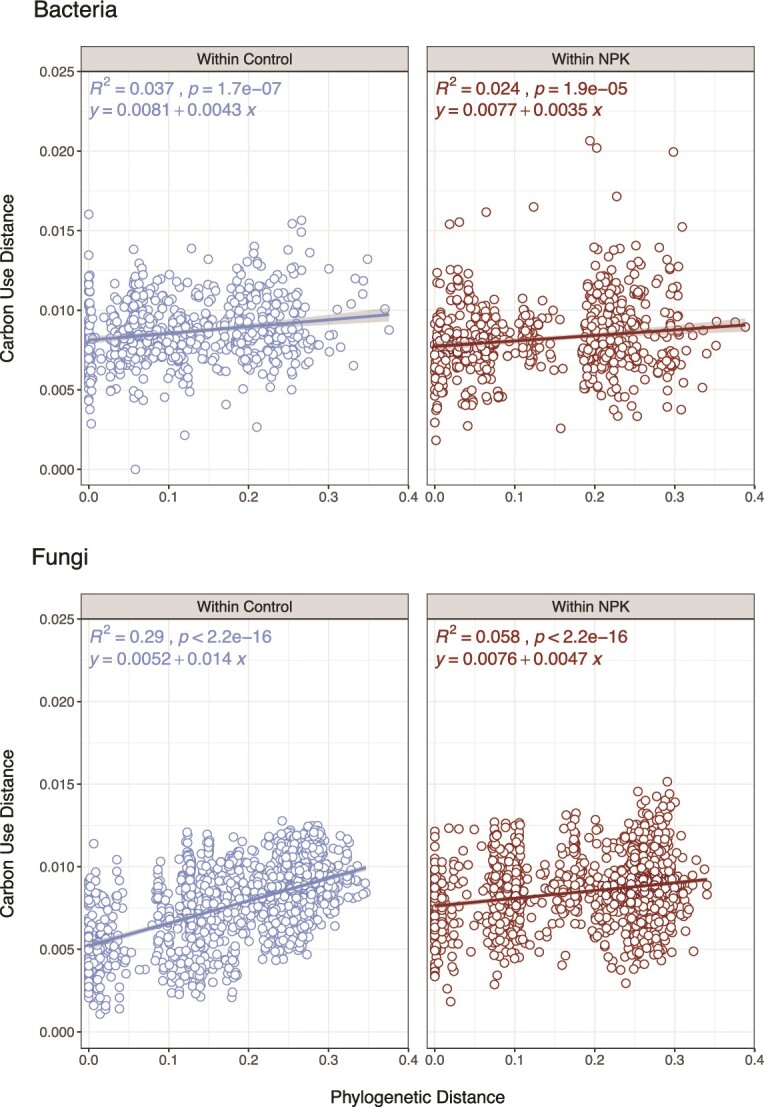
Phylogeny explains a notable percentage of variation in nutrient use distance among fungi from control plots. Scatterplots demonstrating the relationships between the Bray–Curtis dissimilarity of carbon use (*y*-axis) and the pairwise phylogenetic distance (x-axis) for bacterial (A) and fungal (B) foliar endophytes. A linear regression was performed to determine the amount of variance in nutrient use dissimilarity that was explained by phylogenetic distance; best-fit lines are fitted to points for isolates within control or within NPKμ-amended plots and the associated equations are included to highlight the difference in slopes. *R*^2^ values and adjusted *P*-values are indicated for each regression at the top-left corner of each plot. Fungal isolates from control plots demonstrate a strong and significant relationship between phylogenetic distance and carbon use phenotype (*R*^2^ = 0.29; *P* < 2.2e−16).

Among bacterial isolates, phylogenetic distance explained a small but significant amount of variance in carbon use dissimilarity regardless of nutrient status of the plots (NPKu-amended or control) (*R*^2^_B-Control_ = 0.064; *P* = 4.2e−11 and *R*^2^_B-NPKμ_ = 0.026; *P* = 1.2e−06) (Fig. 6A). This result is consistent with the result that resource niche and niche overlap among bacterial endophytes did not appreciably change under nutrient addition treatments. Among endophytic fungi, phylogenetic distance explained 29% of the variance in carbon use among isolates collected from control plots (*R*^2^_F-Control_ = 0.29; *P* < 2.2e−16), but just 6% of this variance among fungi from NPKμ-amended plots (*R*^2^_F-NPKμ_ = 0.058; *P* < 2.2e−16) (Fig. 6B).

The discrepancy in associations between phylogeny and carbon use phenotype among fungi from control and NPKμ-amended plots suggests distinct phylogenetic structures develop within foliar environments depending on soil nutrient status. NPKμ amendment may therefore alter the importance of resource use in shaping fungal phylogeny within leaves, and fungal endophytes are likely further influenced by non-nutrient-related phenotypes and/or resource competition with co-occurring bacteria. In contrast, leaf communities of fungal endophytes in non-amended, control conditions are more strongly defined by phylogenetic relationships among co-occurring fungi that are strongly related to carbon use.

## Discussion

Elevated environmental nutrients can impact the composition and functions of foliar endophytes, with critical implications for large-scale, microbially-driven processes such as nutrient cycling and decomposition [1–4, 21, 29]. However, relatively little is known about the factors mediating foliar endophyte community assembly and function. Here, we report a significant impact of long-term soil NPKμ amendment on the carbon use capacities of foliar endophytes in a long-term field experiment. Our results show that microbial kingdoms respond differently to elevated soil nutrient supply. NPKμ addition altered the carbon use patterns of fungal, but not bacterial, endophytes. Additionally, we inferred patterns in potential resource competitive interactions using our measure of niche overlap, which provides a strong proxy for potential resource competition in a constrained nutrient space. Our findings implicate inter-kingdom resource competition as a possible force that is critical to structuring community assembly, a role that is intensified under NPKμ-amended conditions.

Carbon use phenotypes also exhibited significant associations with microbial phylogenies regardless of soil nutrient status, but the strength of this relationship was notably smaller in fungal communities with chronically elevated nutrients compared to ambient conditions. This further supports our finding that soil nutrient amendment impacts phyllosphere microbial communities and foliar fungi are more strongly affected by these amendments compared to foliar bacteria. Overall, this experiment shows that widespread anthropogenic nutrient deposition via agricultural fertilizer application or industry-driven atmospheric accumulation [15–18] can alter the microbial composition and functional capacities of foliar symbionts. Here, we show the effects of these community-level changes on resource use, growth patterns, and, consequently, the competitive landscape among endophytes within leaves.

NPKμ amendment did not affect the niche width, total growth, and growth efficiency of bacteria compared to control plots. Among fungal endophytes, however, nutrient addition caused changes in the most-utilized C substrates and significant decreases in overall niche width, total growth, and growth efficiency. Fungal isolates from control plots showed high growth efficiency and displayed generalist strategies [57, 58], while fungi from NPKμ-amended plots had lower growth efficiency across smaller resource niche profiles, displaying specialist strategies. While generalist strategies are often favored in spatially or temporally heterogenous environments, specialist strategies develop in stable, unchanging environments [59] or habitats with high degrees of competition among community members [59, 60]. Although temporal and spatial analyses were not pursued in this study, assessing compositional differences among these endophytes across space and time may provide greater insight into the growth trajectories of these communities over ecological and evolutionary time. The heightened specialization observed among endophytic fungi in NPKμ-amended plots may reflect niche differentiation as a response to enhanced resource competition within leaves [43, 44, 61, 62]. Amplified competition for C substrates may stem from the reduced dimensionality of resource niches [63–65], as a high availability of non-C nutrients would increase the strength of competition for C sources among fungi. Foliar fungi show distinct responses to soil nutrient inputs that are not necessarily reflected by their soilborne counterparts [24, 66–68], suggesting an indirect effect of NPKμ on endophytic fungi that is mediated either by the host plant or the broader community within the foliar environment.

These indirect effects of NPKμ on the carbon use patterns of foliar fungi have multiple plausible explanations. First, NPKμ amendment may influence the plant host to impose different environmental filters or selection criteria for specific carbon use profiles among endophytic fungi. Alternatively, non-carbon nutrient inputs may shift the foliar nutrient landscape to be relatively more conducive to bacterial growth, leading to increased bacterial density and higher potential for competitive challenges with fungi in resource space. Indeed, bacterial-fungal (B:F) niche overlap was greater in NPKμ amendment compared to control plots. Wide-scale shifts toward copiotrophic traits have been observed among bacteria in nitrogen- and phosphorus-amended soils [69] and may come at the expense of mycorrhizal fungi and mutualist abundance [66, 67]. Therefore, enhanced bacterial success and competitive pressure toward fungi in leaves are within reason. Finally, carbon use changes among endophytic fungi from NPKμ-amended plots may be related to alternative survival strategies, such as antagonism [70]. Preliminary investigation of fungal inhibition of three bacterial standards suggests marginally greater inhibition among fungal endophytes from NPKμ-amended conditions, though the difference observed was not statistically significant (data not shown). Fungi may respond to enhanced bacterial competition by producing antibiotics, an energetic investment [43, 71] that may reduce growth and/or resource niche size. Indeed, fungi can experience these tradeoffs during heightened resource competition and antagonism toward bacteria [72]. These interactions are shaped by abiotic and biotic factors [45, 73] and can reduce the frequency of intra-kingdom antagonism among fungi [74], which was observed among F:F pairings here. While the discrete mechanisms behind shifts in foliar fungal phenotypes under NPKμ-amended conditions have not yet been fully characterized, endophytic fungi appear to be strongly shaped by the host plant and/or foliar bacterial community rather than the nutrient applications themselves.

Despite limitations associated with phylogenetic analysis using partial 16S and ITS sequences [75, 76] and the advent of approaches performing phylogenetic analyses from metagenomic data [77, 78], our work suggests that the subset of isolates used for phenotyping were representative of all culturable microbes collected. This analysis also indicated that phylogeny was strongly associated with carbon use phenotypes among fungal endophytes from control conditions, explaining 29% of the variance in carbon use. However, the strength of this correlation diminished with NPKμ amendment, and phylogeny explained only 6% of the variance in fungal carbon use under these conditions. Rather, greater inter-kingdom niche overlap and specialist growth strategies among fungi in NPKμ-amended leaves suggest an elevated role of resource use and competition in shaping community assembly in high-nutrient conditions. Specifically, bacterial phenotypes in the leaf environment may constrain phylogenetic and functional diversity of the foliar community [79, 80]. Coupled with potential priority effects [81] and production of secondary metabolites [45], established endophytic bacteria may impart greater competitive pressure on fungi under high nutrient conditions to defend resources in the leaf environment. Therefore, elevated nutrient status may lead foliar fungal communities to be structured more by competitive relationships with foliar bacteria or non-nutrient phenotypes that were not measured here (e.g. species interaction phenotypes or plant colonization-related phenotypes). While we did not assess the potential for homogenization across treatments via aerial movement of microbes between plants (i.e. colonization), the lack of homogenization evidenced by the significant differences between isolates from control and NPKμ-amended plots points to elevated soil nutrient supply as a driving force in mediating endophytic community assembly. This broadly suggests that long-term nutrient deposition fundamentally alters endophyte colonization of leaves, with a distinct shift away from the influence of carbon use phenotypes toward alternative phenotypes of interest.

The long-term consequences of shifting competition between bacterial and fungal endophytes are not entirely clear. Foliar bacteria are important for nutrient uptake, stress modulation, and phytostimulation [82], but are also relatively limited in their abilities to metabolize complex compounds, decompose plant tissue, and cycle nutrients [83–85] relative to fungi. Foliar fungi, however, can metabolize complex substrates and play crucial roles in nutrient acquisition and assimilation [86, 87] as well as decomposition [88], an ecosystem process impacted by high nitrogen deposition [4, 88, 89]. Elevated nutrient supply has been shown to reduce mutualist fungi abundance but enhanced pathogenic fungi abundance and activity [67, 90, 91]. Therefore, nutrient additions may create environments that not only enhance bacterial growth and competitive pressure, but also augment pathogenic fungi at the expense of mutualistic fungi. While it may be difficult to predict specific long-term consequences of such shifts in the inter-kingdom foliar landscape, altered assembly of foliar fungal communities under nutrient deposition is cause for concern for greater ecosystem stability and function.

Collectively, these findings demonstrate that increasing soil nutrient supply can alter carbon use phenotypes and reduce the competitive advantage of foliar fungal endophytes in inter-kingdom resource competition with bacteria. Fungal endophytes in control versus nutrient-amended conditions exhibited generalist and specialist resource use strategies, respectively. Foliar fungi may be indirectly impacted by elevated nutrient supply via altered host plant filtering or modified species interactions with bacteria, mechanisms of community assembly that warrant further research. Future exploration of antagonistic phenotypes among bacterial and fungal endophytes in ambient and nutrient-amended conditions is needed to address these knowledge gaps. Overall, our current results suggest that chronically elevated nutrients fundamentally alter how fungal endophyte communities colonize and persist in plant foliage, with potentially significant consequences for broader ecosystem functions.

## Supplementary Material

Hansen_et_al_ISME_Comms_supplement_revision_final_ycae130

## Data Availability

16S sequences for bacteria and ITS sequences for fungi are available on GenBank (Accessions MW742398–MW742512 and MW751686–MW751805 respectively). Code used to perform statistical analyses and generate visualizations can be found on GitHub (https://github.com/ZoeHansen/PAPER_ISME-Communications_2024).
