# Supplementary material for: Soil nutrient amendment increases the potential for inter-kingdom resource competition among foliar endophytes
Source: ISME Commun. 2024 Oct 25;4(1):ycae130. doi: 10.1093/ismeco/ycae130 (PMC11586052; doi:10.1093/ismeco/ycae130)
Supplement: Hansen_et_al_ISME_Comms_supplement_revision_final_ycae130 [file hansen_et_al_isme_comms_supplement_revision_final_ycae130.pdf]

Supplementary Material

Soil nutrient amendment increases the potential for inter-kingdom resource competition among foliar endophytes

Zoe A. Hansen[1*†], Michael R. Fulcher[2], Nicholas Wornson[1], Seth A. Spawn-Lee[3], Mitch Johnson[4], Zewei Song[1], Matthew Michalska-Smith[1,5], Georgiana May[5], Eric W. Seabloom[5], Elizabeth T. Borer[5], Linda L. Kinkel[1]

[1]Department of Plant Pathology, University of Minnesota, Saint Paul, MN 55108, USA

[2]United States Department of Agriculture, Agricultural Research Service, Foreign Disease-Weed Science Research Unit, Frederick, MD 21702, USA

[3]Department of Integrative Biology, University of Wisconsin-Madison, Madison, WI 53706, USA [Current Address: The Nature Conservancy, Minneapolis, MN 55415]

[4]Department of Horticulture, University of Minnesota, Saint Paul, MN 55108, USA

[5]Department of Ecology, Evolution and Behavior, University of Minnesota, Saint Paul, MN 55108, USA

*Current Affiliation: Biology Department, Carleton College, One North College Street, Northfield, MN 55057, USA

[†]Corresponding author: Zoe Hansen; email: hansen.zoeann@gmail.com; phone: +1 (651) 356-1705; current address: Biology Department, Carleton College, One North College Street, 214 Hulings Hall, Northfield, MN 55057, USA

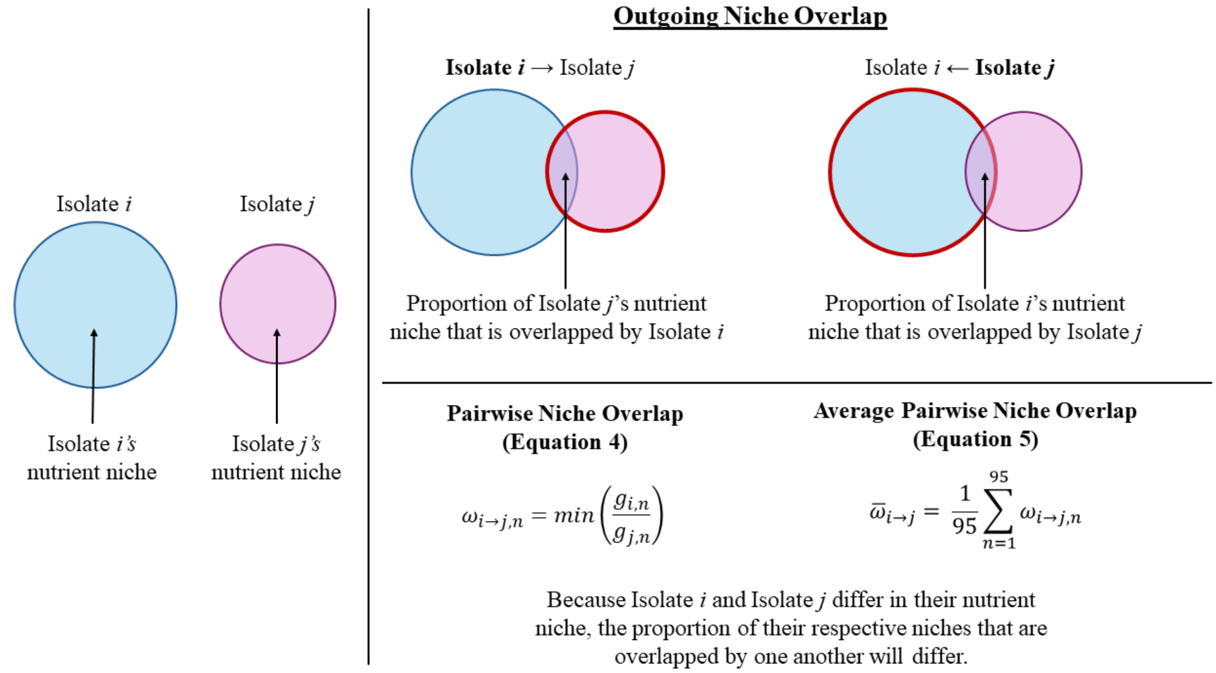

Figure S1. Conceptual display of outgoing niche overlap between two isolates, *i* and *j*. Isolate i and Isolate j have an asymmetrical pairwise relationship in nutrient niche space. While the proportion of Isolate i's niche that is overlapped by Isolate j is relatively small, the proportion of Isolate j's niche that is overlapped by Isolate i is substantially larger. Therefore, the difference in niche size between Isolate i and Isolate j dictates the degree to which each isolate's niche is shared. This difference in niche size also explains why outgoing niche overlap of one isolate ($i \rightarrow j$) is not equal to the outgoing niche overlap of the reciprocal pair ($j \rightarrow i$), as the denominator in Equation 4 would differ.

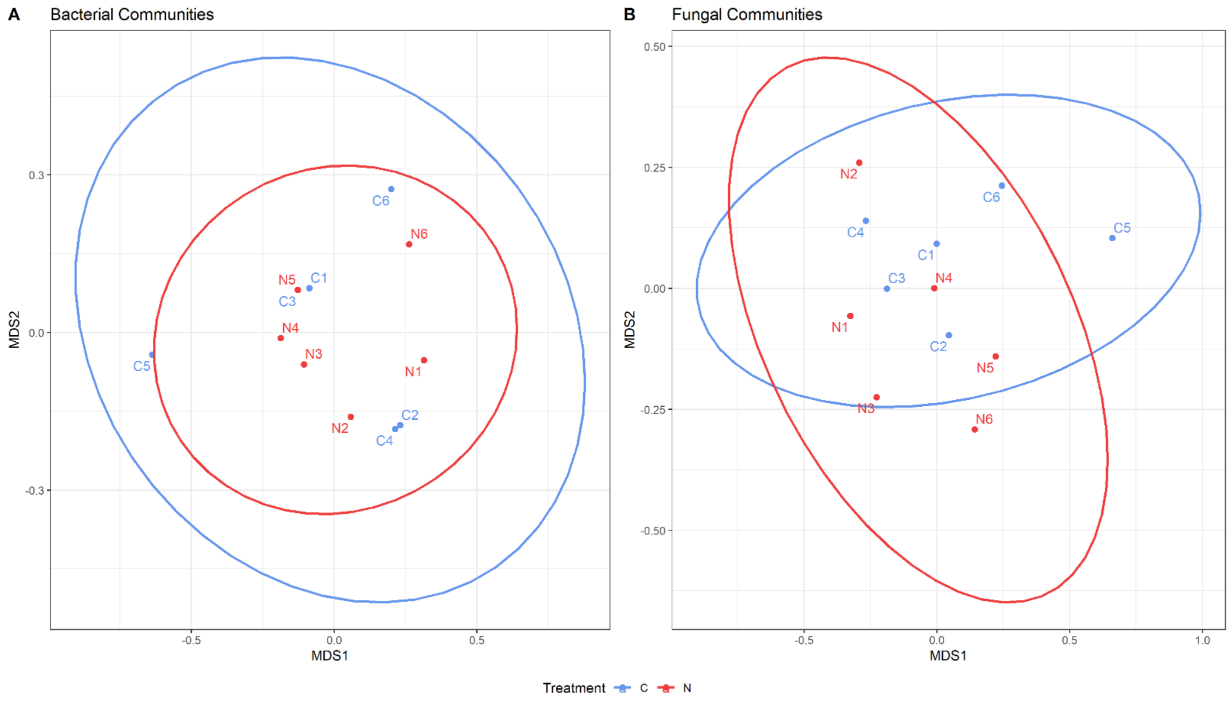

Figure S2. Taxonomic composition of bacterial and fungal endophytes does not significantly differ between soil nutrient supply conditions. Nonmetric multidimensional scaling (NMDS) plots demonstrate similarities and differences in taxonomic composition among bacterial and fungal endophytes between control (blue) and NPKμ-amended (red) plots. Points are oriented based on the Bray-Curtis dissimilarity of taxonomic assignments for all bacterial and fungal isolates included in this study. Each point represents the taxonomic composition of all endophytic isolates collected from a single leaf (n=10 in 11/12 leaves and n=5 bacteria for one leaf). Leaf identities are shown in text adjacent to their associated data point.

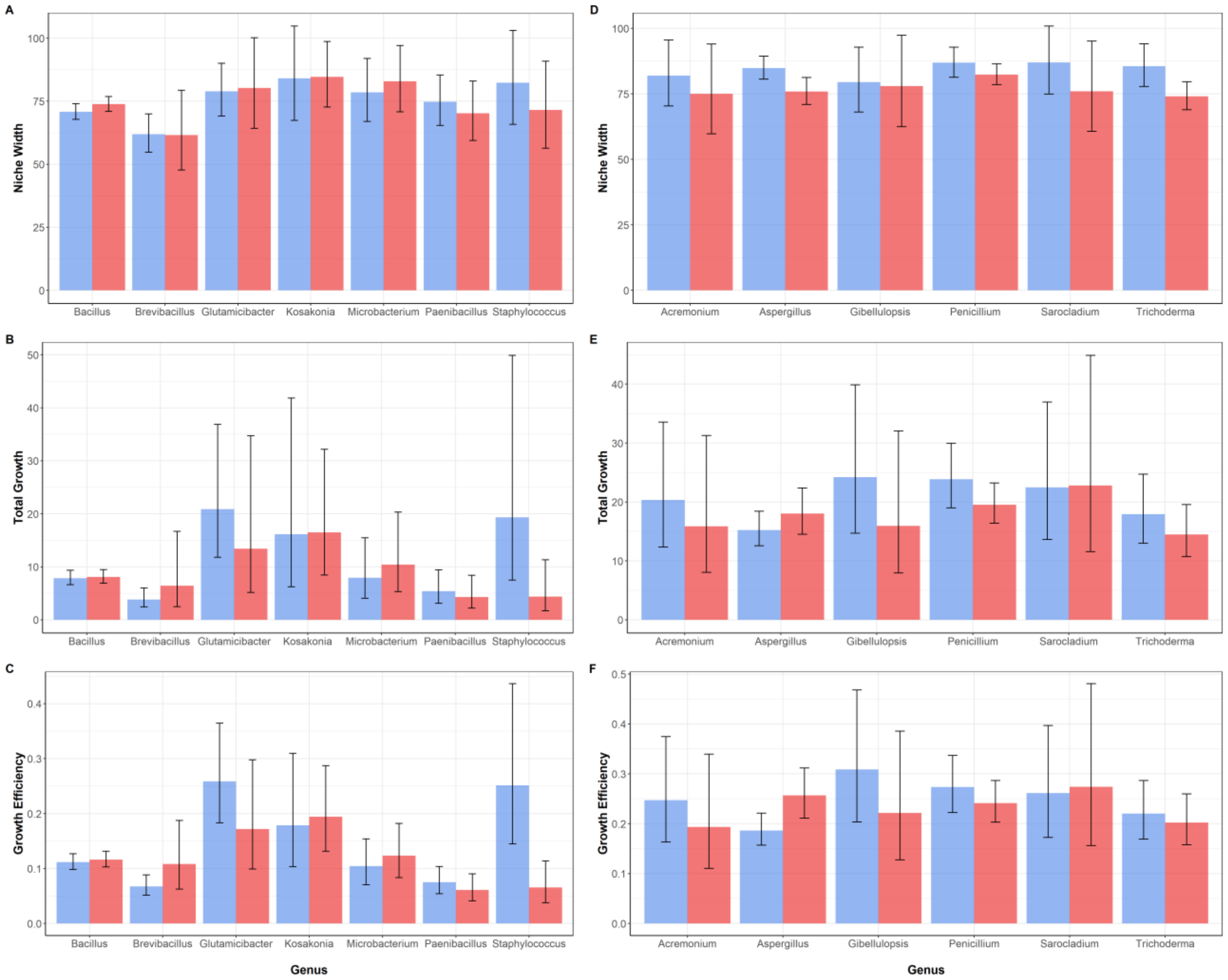

Figure S3. Bacterial and fungal genera present in both control and NPKμ-amended conditions demonstrate coordinated shifts in response to elevated nutrient supply. Bar plots demonstrate the estimated marginal means of three carbon use metrics, niche width, total growth, and growth efficiency, for bacterial (A, B, C) and fungal (D, E, F) endophytes that were collected from both control and NPKμ-amended conditions ("common genera"). Identified genera are shown on the x-axes and associated nutrient metric values are included for endophytes from control (blue) and NPKμ-amended (red) plots. Upper and lower confidence levels are shown as black error bars for each genus.

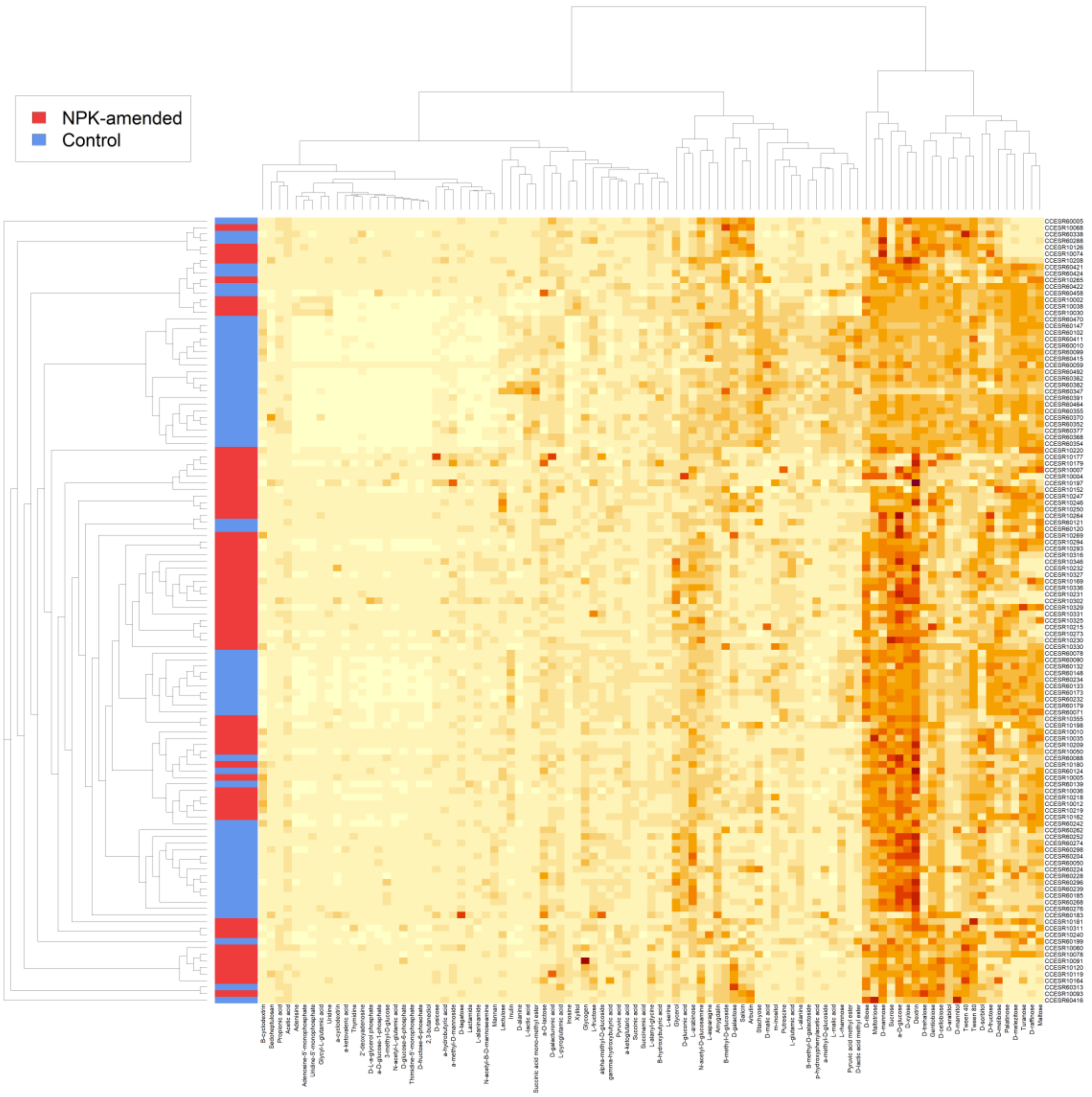

Figure S4. Nutrient use patterns across 95 single-carbon resources do not appear to cluster strongly by phylogeny. A heatmap indicating the total growth (absolute optical density (OD)) of fungal isolates in control and NPKμ-amended conditions. Fungal isolates are shown in rows and nutrients are shown in columns. Fungi from control (blue) and NPKμ-amended (red) plots are ordered according to a RAxML-generated phylogeny using maximum likelihood with random hill climbing and branch support was estimated with bootstrapping via 1000 replicate trees. Nutrients are ordered via hierarchical clustering which displays the similarity of their use among all fungal isolates shown.

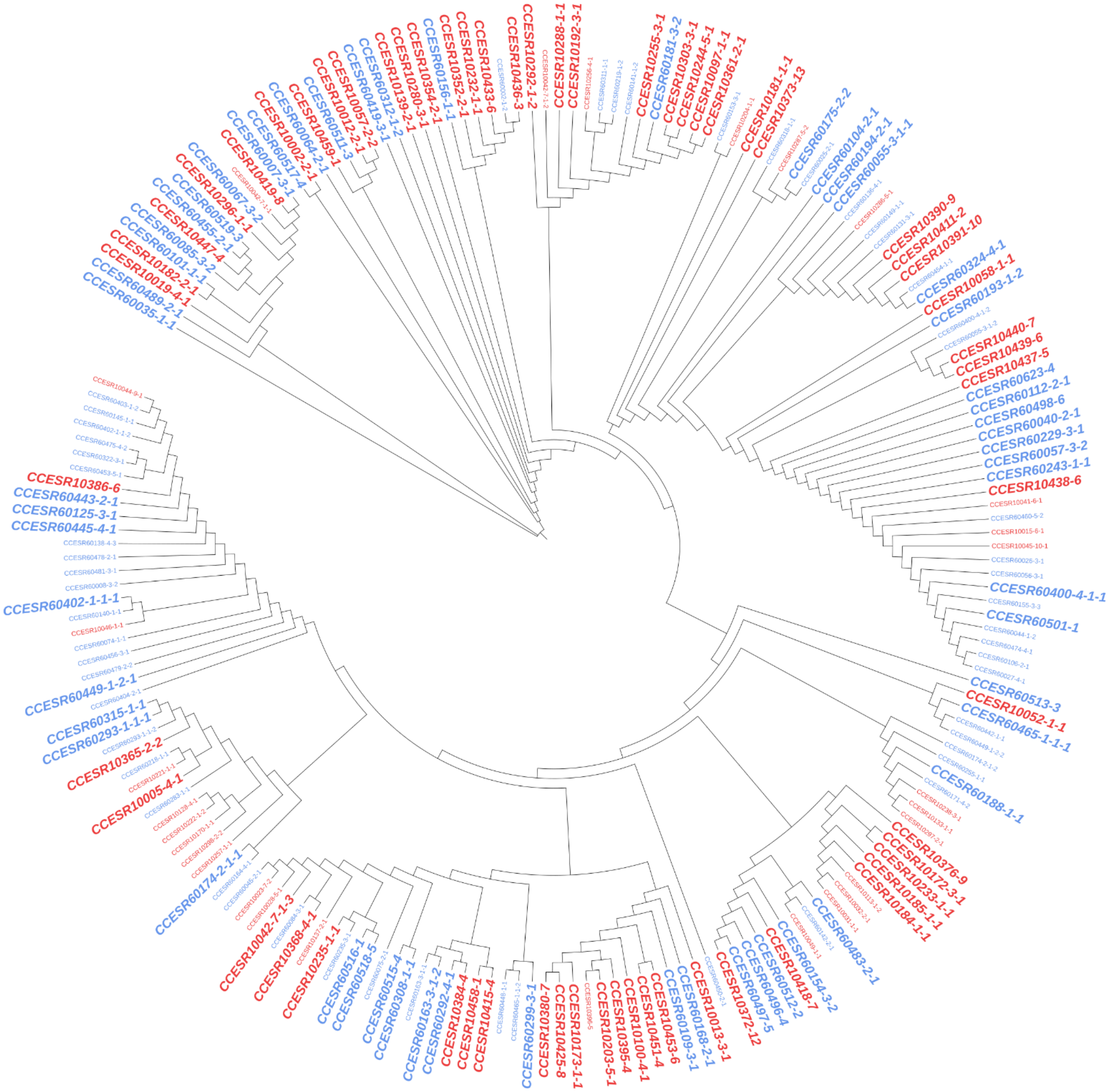
CCESR10440-7
CCESR10439-6
CCESR10437-5
CCESR60623-4
CCESR60112-2-1
CCESR60498-6
CCESR60040-2-1
CCESR60229-3-1
CCESR60057-3-2
CCESR60243-1-1
CCESR10438-6
CCESR10041-6-1
CCESR60460-5-2
CCESR10015-6-1
CCESR10045-10-1
CCESR60028-3-1
CCESR60056-3-1
CCESR60400-4-1-1
CCESR60155-3-3
CCESR60501-1
CCESR10386-6
CCESR60443-2-1
CCESR60125-3-1
CCESR60445-4-1
CCESR60138-4-3
CCESR60478-2-1
CCESR60481-3-1
CCESR60008-3-2
CCESR60402-1-1-1
CCESR60140-1-1
CCESR10046-1-1

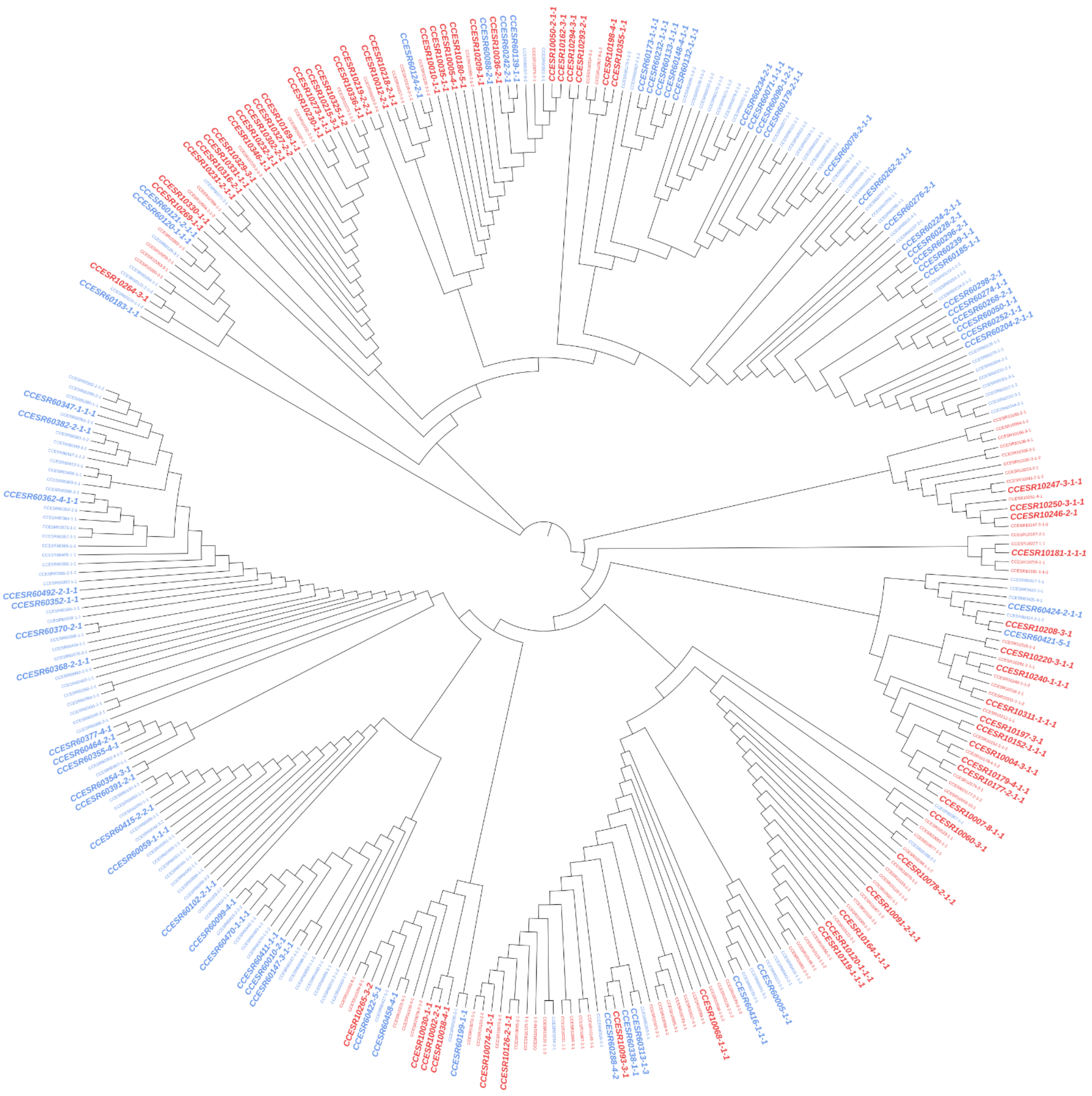

Figure S5. Cultured endophytes included in this study are representative of the set of all cultured isolates collected from *Andropogon gerardii* related to this work. Phylogenetic dendrograms demonstrate the phylogenetic relatedness and distribution among cultured endophytic bacteria (top) and fungi (bottom). Trees were generated using partial gene sequences for the 16S (bacteria) and ITS (fungi) regions. Isolates collected from control conditions are shown in blue while isolates originating from NPKµ-amended conditions are shown in red. The subset of isolates used in this study (n=115 bacteria and n=120 fungi) are designated by bold-italic font with 2x font size. Original branch lengths have been ignored for improved clarity and interpretation.

Supplementary Table S1. Composition of endophytic bacterial and fungal genera among leaves from control and NPKμ-amended plots.

Bacteria	Control	$P_{C\text{-}genus}$[1]	NPK-amended	$P_{NPK\text{-}genus}$[1]	Total: 115
Achromobacter	1	0.02		0.00	1
Atlantibacter		0.00	1	0.02	1
Bacillus	35	0.64	40	0.67	75
Brevibacillus	5	0.09	1	0.02	6
Brevibacterium	1	0.02		0.00	1
Curtobacterium		0.00	3	0.05	3
Glutamicibacter	3	0.05	1	0.02	4
Kocuria	1	0.02		0.00	1
Kosakonia	1	0.02	2	0.03	3
Lysinibacillus	1	0.02		0.00	1
Microbacterium	2	0.04	2	0.03	4
Micrococcus		0.00	1	0.02	1
Paenibacillus	3	0.05	2	0.03	5
Pantoea		0.00	5	0.08	5
Pseudomonas	1	0.02		0.00	1
Rhizobium		0.00	1	0.02	1
Staphylococcus	1	0.02	1	0.02	2
**Fungi**	**Control**	**$P_{C\text{-}genus}$[1]**	**NPK-amended**	**$P_{NPK\text{-}genus}$[1]**	**Total: 120**
Acremonium	2	0.03	1	0.02	3
Aspergillus	17	0.28	11	0.18	28
Cladosporium		0.00	1	0.02	1
Coniochaeta		0.00	1	0.02	1
Daldinia		0.00	5	0.08	5
Didymella		0.00	3	0.05	3
Fusarium	13	0.22		0.00	13
Gibellulopsis	2	0.03	1	0.02	3
Hypoxylon		0.00	1	0.02	1
Macrohyporia	1	0.02		0.00	1
Neocosmospora	7	0.12		0.00	7
Neurospora		0.00	1	0.02	1
Paracremonium	1	0.02		0.00	1
Penicillium	10	0.17	20	0.33	30
Phomatospora		0.00	1	0.02	1
Purpureocillium		0.00	3	0.05	3
Sarocladium	2	0.03	1	0.02	3
Trichoderma	5	0.08	10	0.17	15

Control Only
NPK Only
Both

[1]$P_{C\text{-}genus}$ and $P_{NPK\text{-}genus}$ refer to the proportion of isolates that were assigned to that particular genus in control versus NPK-amended plots, respectively.

Supplementary Table S2. Top-ten consumed carbon substrates for bacterial and fungal endophytes from control and NPKµ-amended plots.

Bacteria					
Control			NPK-amended		
Substrate	$P_{isolates}$[1]	OD_{all}	Substrate	$P_{isolates}$[1]	OD_{all}
L-malic acid*	0.78	0.390	L-malic acid*	0.88	0.432
Dextrin	1.00	0.321	Dextrin	0.98	0.326
D-trehalose	1.00	0.283	D-trehalose	0.98	0.301
D-cellobiose	0.96	0.266	D-mannitol	0.98	0.297
D-mannitol	1.00	0.252	Maltotriose	0.98	0.277
a-D-glucose	0.96	0.239	D-cellobiose	1.00	0.264
D-mannose	0.96	0.239	D-mannose	0.98	0.253
Gentiobiose	1.00	0.236	Gentiobiose	0.98	0.248
Maltotriose	1.00	0.235	a-D-glucose	0.98	0.246
Sucrose°	0.98	0.233	L-asparagine*⁺	0.97	0.245
Average OD		0.269	Average OD		0.289

Fungi					
Control			NPK-amended		
Substrate	$P_{isolates}$[1]	OD_{all}	Substrate	$P_{isolates}$[1]	OD_{all}
Dextrin	1.00	0.770	a-D-glucose	1.00	0.807
a-D-glucose	1.00	0.765	Dextrin	1.00	0.777
D-mannose	1.00	0.745	D-mannose	1.00	0.701
D-xylose^	1.00	0.682	D-xylose^	1.00	0.683
Maltotriose	1.00	0.646	Sucrose	0.98	0.619
Sucrose	1.00	0.630	D-ribose^⁺	0.98	0.591
D-melezitose^°	1.00	0.592	Maltotriose	1.00	0.576
Tween 80°	1.00	0.579	Gentiobiose⁺	1.00	0.533
Maltose°	1.00	0.572	D-cellobiose	1.00	0.520
D-cellobiose	1.00	0.567	D-trehalose⁺	1.00	0.483
Average OD		0.655	Average OD		0.629

Substrate class	Symbol key
Carbohydrates	* Bacteria-only
Polymers	^ Fungi-only
Carboxylic Acids	° Control-only (within Kingdom)
N-Source	⁺ NPK-amended-only (within kingdom)

[1]$P_{isolates}$ refers to the proportion of total isolates in a particular group (e.g., Bacteria from plants in control plots) that consumed the substrate.
